# A Pilot Study of F-18 Fluciclovine-PET/CT as a Diagnostic Tool for Bone Metastases in Patients With Castrate Resistant Prostate Adenocarcinoma and Correlative Analysis of Blood and Bone Molecular Testing (The FACT Study)

**DOI:** 10.1093/oncolo/oyad242

**Published:** 2023-08-24

**Authors:** Hani M Babiker, Matthew D Kay, Carol Stuehm, Gregory Woodhead, Phillip H Kuo

**Affiliations:** Department of Medicine, Division of Hematology-Oncology, Mayo Clinic Cancer Center, Mayo Clinic Florida, Jacksonville, FL, USA; Department of Medical Imaging, University of Arizona, Tucson, AZ, USA; Department of Medical Imaging, University of Arizona, Tucson, AZ, USA; Department of Medical Imaging, University of Arizona, Tucson, AZ, USA; Departments of Medical Imaging, Medicine, and Biomedical Engineering, University of Arizona, Tucson, AZ, USA

## Abstract

**Background:**

Suspicious F-18 fluciclovine PET/CT findings for osseous metastases from prostate cancer (PC) were targeted for core needle biopsy. We correlated the maximum standardized uptake value (SUVmax) of biopsied lesions, with biopsy results, other diagnostic outcomes, and blood and tissue molecular analysis (TMA).

**Material and Methods:**

Patients with castrate resistant prostate cancer (CRPC) were recruited from a university oncology clinic. SUVmax, histology, blood, and TMA were correlated.

**Results:**

Fifteen patients were enrolled and 12 underwent bone biopsies. Fifty percent of bone biopsies demonstrated malignancy. Higher SUVmax was associated with positive biopsies for adenocarcinoma (*P* = .003), and lesions with SUVmax ≥ 5.1 were all positive for malignancy. Significant correlation between blood and somatic TMA (*P* = .002) was also found.

**Conclusion:**

Higher uptake of F-18 fluciclovine was associated with higher predictive value for osseous metastasis on biopsy. There was a significant correlation between blood and TMA.

## Teaser

This study provides a valuable model for amino acid PET, as well as important molecular data on tumors. ctDNA correlates with tissue next-generation sequencing in prostate cancer.

## Introduction

Prostate cancer (PC) is the most common cancer in men, with a high cancer-related death in patients with advanced disease.^1^ Anti-1-amino-3-^18^F-flurocyclobutane-1-carboxylic acid (^18^F-fluciclovine) is a synthetic amino acid PET radiotracer approved by the FDA for the detection of recurrent PC.^2^ Although data supporting accuracy of F-18 fluciclovine PET/CT for lymph node (LN) disease is strong, scant data exist for bone metastases.^3^ For LN disease, F-18 fluciclovine PET/CT demonstrated high positive and negative predictive values (PPV, NPV) with increasing PSA levels. A PSA of >3.98 to ≤ 8.9 was associated with a PPV and NPV of 95% and 100%, respectively.^3^ PSA levels of >8.9 were associated with a PPV and NPV of 100% each. Most patients with CRPC present with bone metastases, and exploring the role of F-18 fluciclovine PET/CT as a diagnostic tool for PC with bone metastases may provide important molecular data on tumors.^4^ Given the need for bone biopsy to confirm recurrence in some cases and the increased use of TMA to identify targetable mutations, we assessed the validity of F-18 fluciclovine PET/CT for directing CT-guided biopsy, then assessed the correlation of circulating tumor DNA (ctDNA) and TMA. Although prostate-specific membrane antigen positron tomography (PSMA PET) has surpassed F-18 fluciclovine PET/CT as a diagnostic tool in PC, we sought to model this study for future use on amino acid PET which is currently being studied in different malignancies. Other studies have evaluated ctDNA and TMA in patients with PC; however, our study was conducted in heavily pre-treated patients referred for a targeted mutation-match early phase clinical trial and hence we sought to investigate reliability of ctDNA.

## Material and Methods

The FACT study was conducted in accordance with the International Council on Harmonisation E6 requirements for Good Clinical Practice and with the ethical principles of the Declaration of Helsinki. Written informed consent was provided to all patients prior to study initiation (NCT03496844). The primary goal was validation by core needle of possible osseous metastases identified on F-18 fluciclovine PET/CT and the secondary aim was assessing the correlation between ctDNA and TMA mutation concordance rate (mCR). The mCR was assessed as common mutations/all mutations (genes) of each panel (ctDNA and TMA) and Spearman was used to assess ratios correlation. NGS panel used is described in the Supplementary Material. Patients with CRPC in need of biopsy for TMA were included. Following the intravenous injection of approximately 10 mCi of F-18 fluciclovine, CT was performed at low dose without intravenous contrast. After an uptake period of approximately 3 minutes, PET imaging was performed from pelvis to vertex of skull and was iteratively reconstructed with time of flight.

### Statistical Analysis

Analyzed prospective and retrospective data were described descriptively. Sensitivity, specificity, true positive, and false positive rates findings on F-18 fluciclovine PET/CT compared to bone biopsies were calculated. Spearman correlation was used to study correlation between the mutation concordance rate of ctDNA and TMA.

## Results

### Demographics

Fifteen patients were enrolled into the study, age range was 53-87 years, and all patients had stage IV prostate adenocarcinoma. Demographic data are presented in Table 1. 33.3% of patients had soft-tissue metastasis and 86.6% of patients had bone metastasis by F-18 fluciclovine. One patient had biopsies of both bone and soft tissue. Eighty percent of patients underwent bone biopsy with the iliac bone as a target in 75% of those cases, and 50% of bone biopsies showed adenocarcinoma. 26.6% soft tissue biopsies were performed and all showed adenocarcinoma.

**Table 1.** Patient demographics.

Age range (years):	53-87 (m 70.4).
Gleason score range	6-9, 47% of pts had a GS of 9
PSA range	1.8-4476.8 (m 430.4)
Soft tissue metastasis (pts)	33.3%
Bone metastasis (pts)	86.6%
Castrate resistance disease (pts)	93.3%
High-risk hormonal sensitive disease (pts)	6.6%
*Prior treatments (pts)*	
1.Docetaxel	26.6%
2.Enzalutamide	66.6%
3.Abiraterone	53.3%
4.Oral anti-androgens	66.6%
5.ADT	100%
ALK-P range	16-2605 IU/L (m 255.8)
LDH range	133-623 IU/L (m 267)
HGB range	9.3-16.6 g/dl (m 13.2)

Abbreviations: ADT: androgen deprivation therapy; ALK-P: alkaline phosphatase; HGB: hemoglobin; LDH: lactate dehydrogenase; m: mean; pts: patients.

### Axumin PET Scan Analysis

F-18 fluciclovine PET/CT was used to identify biopsied lesions in all cases. The SUV max range of biopsied lesions was 1.6-12.8 and the median SUV max was 7. The median SUV max of biopsy-positive lesions and biopsy-negative lesions were 8.7 and 4.7, respectively. The difference in the mean SUV max of the biopsy-positive and negative lesions was significant (*T* = 3.27656, 8.4 and 4.75, *P-*value = .002757). Lesions with an SUV max >5.1 were all positive lesions on biopsy (Fig.s 1i&ii A and B). The calculated sensitivity and specificity of F-18 fluciclovine in bone biopsy were 78% and 40%, respectively. The PPV and NPV were 54% and 67%, respectively. Negative biopsies were confirmed by demonstrating a minimum of 12 months stability on follow-up imaging (note that follow-up was not available for 2 patients, so these negative biopsies were presumed to be true negatives).

**Figure 1:**
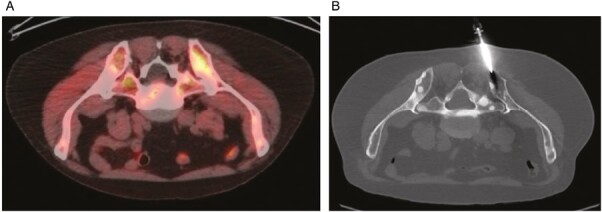

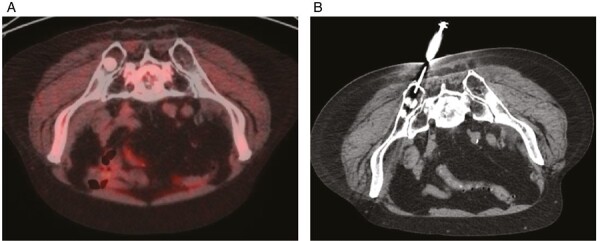
F-18 fluciclovine PET with a biopsy-positive bone metastasis. (**iA**) Fused transaxial fluciclovine-PET/CT image demonstrates asymmetrically increased uptake in the left posterior iliac bone with SUVmax 6.8. Image is flipped to simulate prone position for easier comparison to biopsy position. (**B**) Transaxial image from CT performed for image-guided biopsy in prone position shows the successful placement of the needle. Pathology confirmed metastatic prostate adenocarcinoma. F-18 fluciclovine PET with a biopsy-negative bone metastasis. Fused transaxial fluciclovine-PET/CT image demonstrates a sclerotic lesion suspicious for metastasis in the right posterior iliac bone with SUVmax 1.6. Image is flipped to simulate prone position for easier comparison to biopsy position. (**B**) Transaxial image from CT performed for image-guided biopsy in prone position shows the successful placement of the needle. Pathology did not reveal any malignant cells.

### ctDNA and TMA Results

All patients had ctDNA analysis (Guardant360) and a driver/targetable mutation was identified in 67% of patients. The most common alterations were AR (47%), TP53 (27%), MYC amplification (20%), PIK3C (13%), and ATM/BRCA2 (6.7% each). Fifty percent of patients (6/12) who had bone biopsies underwent TMA (CARIS), such that 40% of the total number of patients (6/15) had TMA results. The most common reason that TMA was not performed was inadequate sample. A targetable alteration was found in 40% out of the total number of patients in the study, and 100% of the patients who had bone biopsies with positive histology for adenocarcinoma underwent TMA testing. The most common TMA alteration was AR (100%) and other TMA alterations identified were BRCA2, FGF3 amp, CDK12, SF3B1, and ARV7 (17% each). Two patients had positive germline testing, one had a CHEK2 and BRCA2 mutations and was treated with Olaparib and achieved a 93% reduction in PSA. The other patient had a BRCA2 mutation and was treated with a targeted drug and had a PSA reduction by 99.8% and a partial response on CT. The Spearman correlation between the ctDNA and TMA mutation concordance rate was significant (*R*^2^ = 0.9, *P* (2-tailed) = .00192).

## Discussion

F-18 fluciclovine PET/CT demonstrated utility for localizing recurrent disease in patients with biochemical relapse of prostate cancer after definitive treatment and mostly in lymph node recurrence. F-18 fluciclovine was demonstrated to accumulate in both osteolytic and osteoblastic lesions in addition to early-stage osteoblastic bone metastases.^5^ TMA analysis is increasingly critical for treatment decisions, thus the need to explore its role in CRPC with osseous metastases, as the most common site for metastasis, is paramount. Our study demonstrates the benefit of utilizing F-18 fluciclovine PET/CT to localize bone lesions for histologic confirmation, specifically in lesions with an SUV max of >5.1, and showed positive sensitivity, specificity, PPV, and NPV, albeit in a small number of patients. Our trial demonstrated a significant Spearman correlation in the mutation concordance rate between ctDNA and TMA indicating a potential role for ctDNA in identifying targetable alterations as bone biopsy is commonly low yield, invasive, and difficult for patients with PC. This is essential given the role of targeted therapy for standard of care treatment or clinical trials.

## Supplementary Material

oyad242_suppl_Supplementary_Material
