# Supplementary material for: A Pilot Study of F-18 Fluciclovine-PET/CT as a Diagnostic Tool for Bone Metastases in Patients With Castrate Resistant Prostate Adenocarcinoma and Correlative Analysis of Blood and Bone Molecular Testing (The FACT Study)
Source: Oncologist. 2023 Aug 24;28(11):e1114–7. doi: 10.1093/oncolo/oyad242 (PMC10628580; doi:10.1093/oncolo/oyad242)
Supplement: oyad242_suppl_Supplementary_Material [file oyad242_suppl_supplementary_material.docx]

**Supplementary Material:**

NGS Panel utilized:

ctDNA was extracted from plasma, genomic alterations were analyzed by parallel sequencing of amplified target genes (73 genes) using Illumina Hi Seq (Guardant360). Direct sequence analysis was performed on genomic DNA isolated from FFPE TT using the Illumina MiSeq platform, 592-whole gene targets (Caris MI/X).

Specificity and sensitivity:

The definition of specificity is “the percentage of people who test negative for a specific disease among a group of people who do not have the disease”. Sensitivity may describe how well a test can detect a specific disease or condition in people who have the disease or condition. For this specific trial: The trial obtained correlative F-18 fluciclovine-PET imaging and bone biopsy results in patients with advanced prostate cancer. Patients with negative biopsies were followed for minimum of another 12 months (standard of care imaging and clinical assessment) for possibility of false negative biopsy (specificity). Sensitivity was based on positive uptake on the F-18 fluciclovine-PET and positive biopsy over total patients who had positive uptake on the scan. The study calculated true positive and false positive rate of positive bone finding (positive histology demonstrating malignancy) on F-18 fluciclovine -PET scan compared to gold standard of bone biopsy.

Positive and negative concordance rate: The secondary aim of the study was to evaluate Spearman correlation between individual mutations concordance rate ratios between ctDNA and TMA. However, the positive and negative concordance rate (total number of concordant pairs/total number of pairs) was 50% each.
